# Long non-coding RNA H19 protects against intracerebral hemorrhage injuries via regulating microRNA-106b-5p/acyl-CoA synthetase long chain family member 4 axis

**DOI:** 10.1080/21655979.2021.1951070

**Published:** 2021-07-21

**Authors:** Bing Chen, Haoran Wang, Chenglin Lv, Chongdan Mao, Yuguang Cui

**Affiliations:** Department of Neurosurgery, Qingdao Eighth People’s Hospital, Qingdao, Shandong, China

**Keywords:** Intracerebral hemorrhage, ferroptosis, lncRNA H19, miR-106b-5p, acyl-CoA synthetase long-chain family member 4

## Abstract

Intracerebral hemorrhage (ICH) is one of the most common refractory diseases. Long non-coding RNAs (lncRNAs) play crucial roles in ICH. This study was designed to investigate the role of lncRNA H19 in ICH and the underlying molecular mechanisms involved. Real-time quantitative polymerase chain reaction (RT-qPCR) was performed to determine mRNA expression. Cell viability was analyzed using Cell Counting Kit 8 (CCK8). PI staining Flow cytometry and TdT-mediated biotinylated nick end-labeling (TUNEL) assays were performed to determine ferroptosis in brain microvascular endothelial cells (BMVECs). Targeting relationships were predicted using Starbase and TargetScan and verified by RNA pull-down and luciferase reporter gene assays. Western blotting was performed to assess protein expression. LncRNA H19 is highly expressed in ICH model cells. Over-expression of H19 suppressed cell viability and promoted ferroptosis of BMVECs. miR-106b-5p is predicted to be a target of H19. The expression of miR-106b-5p was lower in oxygen and glucose deprivation hemin-treated (OGD/H-treated) cells. Over-expression of miR-106b-5p reversed the effects of H19 on cell viability and ferroptosis in BMVECs. Furthermore, acyl-CoA synthetase long-chain family member 4 (*ACSL4*) was verified to be a target gene of miR-106b-5p and was highly expressed in OGD/H-treated cells. Upregulation of ACSL4 inhibited the effects of miR-106b-5p and induced BMVEC dysfunction. In conclusion, lncRNA H19 was overexpressed in ICH. Knockdown of H19 promoted cell proliferation and suppressed BMVECs ferroptosis by regulating the miR-106b-5p/ACSL4 axis. Therefore, H19 knockdown may be a promising therapeutic strategy for ICH.

## Graphical abstract

**Figure:**
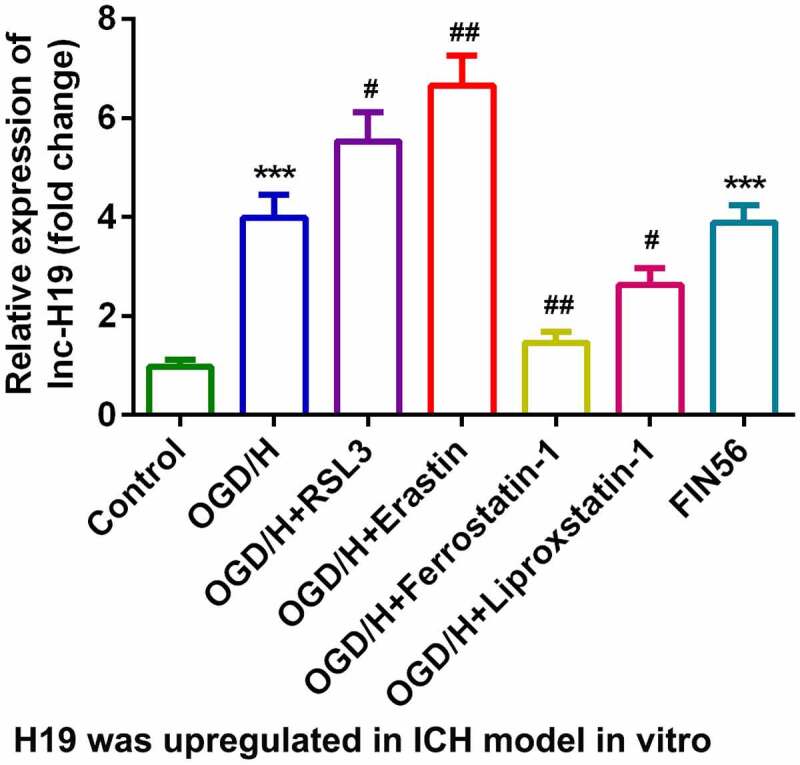

## Introduction

Intracerebral hemorrhage (ICH) is a common and frequently occurring disorder characterized by high rates of morbidity, mortality, and disability [1,2]. After the occurrence of ICH, active substances released by fusion of the hematoma lead to progressive damage to brain tissues [3,4]. The degree of ischemic injury to brain tissue around the hematoma directly affects patient prognosis and outcome [5].

Recent studies have indicated that the iron content released by erythrocyte lysis in hematomas after ICH is consistently higher than normal, which is an important factor causing secondary brain injury [6,7]. Ferroptosis is a regulated cell death (RCD) process caused by redox state disorder of the intracellular microenvironment controlled by glutathione peroxidase 4 (GPX4) due to intracellular iron overload [8]. Ferroptosis is accompanied by changes in glutathione (GSH), iron content, lipid peroxidation (LPO), and malondialdehyde (MDA) [9–11]. Emerging evidence shows the potential of ferroptosis therapy in liver disease [12], cancer [13,14] and brain disorders [15]. The initiation and progression of ICH are closely related to ferroptosis of nerve cells after ICH [16,17]. Therefore, understanding the mechanisms involved in nerve cell death after ICH and taking appropriate intervention measures to inhibit ferroptosis during the disease course are key to reducing secondary brain injury.

Long non-coding RNAs (lncRNAs) are members of the non-coding RNA family with a length of > 200 nt that function as a sponge and regulate various diseases by competitively binding to micro RNAs (miRNAs) [18,19]. miRNAs further target downstream genes [20]. Kim et al. found that the expression of lncRNA H19 is significantly upregulated in ICH [21]. Furthermore, H19 plays essential roles in the regulation of angiogenesis, adipocyte differentiation, lipid metabolism, inflammatory responses, cellular proliferation, and apoptosis [22]. However, its role in ICH remains poorly understood. In the present study, we sought to elucidate H19 regulatory pathways in ICH.

In this study, lncRNA H19 was found to be upregulated in ICH injury *in vitro*. Suppression of H19 in oxygen and glucose deprivation hemin-treated (OGD/H) ICH model cells promoted cell viability and inhibited ferroptosis. Meanwhile, downregulation of miR-106b-5p, which is a target of H19, reversed the effects of H19 on cellular functions. Therefore, there is an urgent need to investigate whether the regulatory roles of H19 in ICH could be mediated by sponging miR-106b-5p.

## Materials and methods

### Cell culture and transfection

Brain microvascular endothelial cells (BMVECs) obtained from the Institute of Hematology, Chinese Academy of Medical Sciences, were used to establish a model of ICH in *vitro*. Cells were cultured in glucose- and serum-free dulbecco’s modified eagle medium (DMEM; EuroClone, Milan, Italy) under 5% CO_2_ and 95% N_2_ for 10 min. The cells were then cultured with hemin (10 μM, Sigma, New Jersey, USA) and maintained for 2 h in an anaerobic chamber filled with 5% CO_2_ and 95% N_2_ and restored back to normal culture conditions for oxygen and glucose deprivation (OGD) termination [23].

Transfections [23] were performed when cell confluence reached approximately 60%. The H19 inhibitor plasmid, miR-106b-5p mimic/inhibitor plasmid, OE-ACSL4 vector, and their negative controls (all obtained from Sangon, Shanghai, China) were diluted to a final concentration of 20 μM, and then 5 μl Lipofectamine® 3000 reagent (Thermo Fisher Scientific, Califonia, USA) were combined with 5 μl transfection plasmids, and incubated for 20 min at room temperature under the guidance of the manufacturer’s instruction. After 6 h of transfection, the solution was replaced with DMEM (EuroClone, Milan, Italy).

### Real-time quantitative polymerase chain reaction (RT-qPCR)

RT-qPCR was used to measure mRNA and miRNA levels. BMVECs were mixed with TRIzol® reagent (Thermo Fisher Scientific) to extract total RNA. Reverse transcription and qPCR were carried out using a BlazeTaq One-Step SYBR Green RT-qPCR Kit (with ROX) (QP071; GeneCopoeia, Maryland, USA) on a SEDI Thermo Cycler controlled by Control Bus Net software package (Wealtec Bioscience, Taiwan, China). The RT-qPCR conditions consisted of initial denaturation at 95°C for 30 s, followed by 40 cycles of denaturation at 95°C for 5 s and annealing at 60°C for 30 s. Conditions for the melt-curve analysis were one cycle of denaturation at 95°C for 10 s followed by an increase in temperature from 65°C to 95°C at a rate of 0.5°C/s. All primers were designed and synthesized by Nanjing Genscript Biotech Co., Ltd. (Jiangsu, China), and *GAPDH* and *U6* were used as internal references. -Fold changes in the indicated genes were calculated using the 2^−ΔΔCt^ method [24]. The primer sequences were as follows:

H19: (Forward): 5ʹ-GCACCTTGGACATCTGGAGT-3ʹ;

(Reverse): 5ʹ-TTCTTTCCAGCCCTAGCTCA-3ʹ;

miR-106b-5p: (Forward): 5ʹ-TGCGGCAACACCAGTCGATGG-3ʹ;

(Reverse): 5ʹ-CCAGTGCAGGGTCCGAGGT-3ʹ;

*GAPDH*: (Forward): 5ʹ-AGGTGAAGGTCGGAGTCAACG −3ʹ;

(Reverse): 5ʹ-AGGGGTCATTGATGGCAACA-3ʹ;

*ACSL4*: (Forward): 5ʹ-CATCCCTGGAGCAGATACTCT-3ʹ;

(Reverse): 5ʹ-TCACTTAGGATTTCCCTGGTCC-3ʹ;

*U6*: (Forward): 5ʹ-CTCGCTTCGGCAGCACATATACT-3ʹ;

(Reverse): 5ʹ-ACGCTTCACGAATTTGCGTGTC-3ʹ;

### Cell counting kit 8 (CCK8)

Cell viability was determined using CCK8 assays [25]. Cells were resuspended at 1 × 10^5^ cells/ml and then placed into 96-well plates (100 μl/well). Ten microliters of CCK8 reagent (AMJ-KT0001; AmyJet Technology, Beijing, China) was added to each well of the plate and cultured in an incubator at 37°C for 4 h to detect cell survival. Optical absorbance values were evaluated using a microplate reader (HBS-1096 C; Nanjing DeTie Experimental Equipment, Jiangsu, China) at a wavelength of 450 nm.

### Fluorescent staining

PI staining [26] was used to determine cell death of BMVECs. Briefly, after transfection, cells were collected and suspended. Afterward, cells added with 10 µL of PI reagents (Vazyme Biotech, Jiangsu, China). Cells were counterstained with DAPI. Subsequently, cells were visualized using a fluorescent microscope (Nikon Tokyo, Japan).

Death of BMVECs was measured using an *in situ* cell death detection kit (11684817910, Roche, Basel, Switzerland) as previously described [27]. In brief, cells were deparaffinized, permeabilized with 0.1% Triton X-100 (ST795, Beyotime Biotech, Jiangsu, China), and incubated with 0.3% H_2_O_2_. After washing with PBS, cells were incubated with terminal dexynucleotidyl transferase mediated biotinylated nick end-labeling (TUNEL) reaction mixture at 37°C for 1 h, following the manufacturer’s protocol. After that, the cells were incubated with terminal dexynucleotidyl transferase (TdT) reaction cocktail at 37°C for 30 min. After washing with PBS three times, the cells were stained with hematoxylin for 3 min. Finally, cell images were captured using a fluorescent microscope.

### Luciferase reporter gene assay

The interaction between miR-106b-5p and H19 or ACSL4 was determined using luciferase reporter gene assay as previously described [28]. Briefly, wild-type and mutated 3ʹ-UTR regions of H19 and ACSL4 luciferase reporter gene vectors were designed and synthesized by Guangzhou RiboBio Co., Ltd., China. The cells were transfected with miR-106b-5p mimic or miR-NC mimic and the wild-type or mutant of H19 or ACSL4 for 48 h. Cells were then lysed to detect luciferase activity using a luciferase reporter gene assay kit (K801-200; BioVision Tech, San Francisco, USA). Luciferase activity was normalized to Renilla luciferase activity.

### RNA pull-down assay

RNA pull-down assays were carried out using the MagCapture RNA Pull Down Assay Kit (297–77501; Whatman, Metestone, UK), according to the manufacturer’s instructions [29]. Briefly, cells were lysed and incubated with a biotinylated miR-106b-5p probe and its negative control. Streptavidin-labeled magnetic beads were resuspended and cultured with the probes (50 pmol) at 4°C overnight. Next, the beads were eluted from the RNA-protein complexes. The results were determined using RT-qPCR.

### Western blot assay

RIPA reagent (Sigma-Aldrich, New Jersey, USA) was used to extract proteins from BMVECs [23]. Protein concentration was determined using a BCA kit (Sigma-Aldrich). Additionally, proteins (20 µg/lane) were separated by 15% SDS-PAGE and then transferred to PVDF membranes (Bio-Rad, California, USA). The membranes were blocked with 5% skim milk for 2 h. Membranes were then incubated with primary antibodies, including SLC7A11 (ab175186, 1: 3000, Abcam, California, USA), GPX4 (ab125066, 1: 2000, Abcam), TRF1 (ab129177, 1: 1000, Abcam), anti-ACSL4 (ab205199, 1:1000, Abcam), and mouse anti-GAPDH (ab8245, 1: 500, Abcam) at 4°C overnight, followed by incubation with secondary goat anti-mouse antibody against immunoglobulin G (IgG; ab205719, 1: 2000, Abcam), and goat anti-rabbit antibody against IgG (ab6721, 1:2000, Abcam) for 1 h. Subsequently, the membrane was stained with an Electrochemiluminescence (ECL) western blotting kit (ab193759, Abcam). GAPDH was used as loading control. Finally, protein bands were visualized using an ECL system (Thermo Fisher Scientific).

### Statistical analysis

SPSS v.19.0 (IBM, Newyork, USA) was used for statistical analyses. All experiments were performed in triplicate and all data are presented as means ± SD. Differences between two groups were evaluated using Student’s *t*-test and one-way ANOVA for multiple groups. Statistical significance was set at *P*< 0.05.

## Results

### H19 was highly expressed in the OGD/H model group with ferroptosis

**Figure 1.**
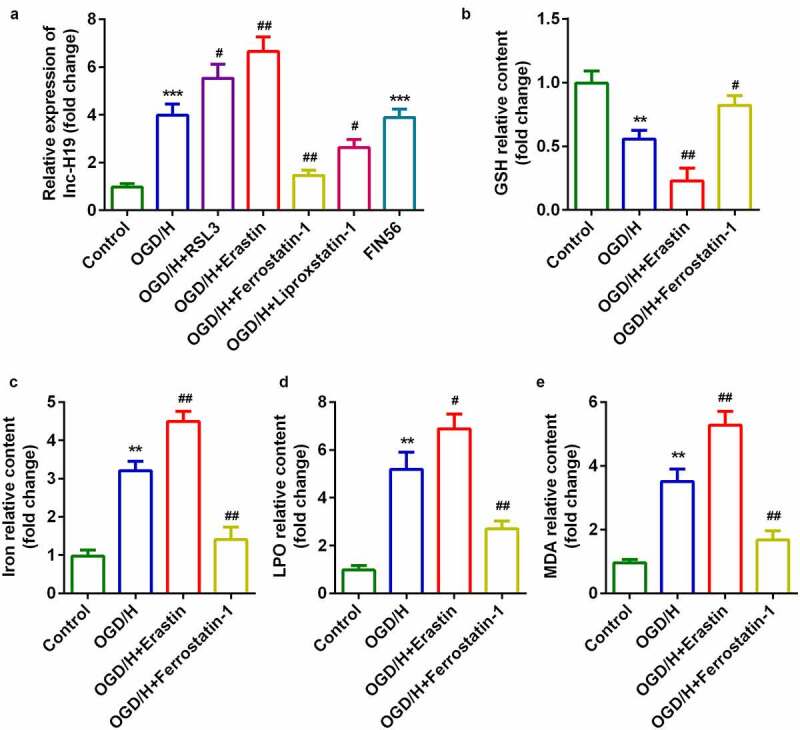
H19 was highly expressed in the OGD/H ICH model cells

To explore the role of H19 in ICH progression, the expression of H19 was detected by qPCR. In the OGD/H model and ferroptosis activator (FIN56) groups, the expression level of H19 was sharply increased compared to that in the control group (Figure 1(a)). The expression level of H19 was significantly increased by the addition of the ferroptosis activators RSL3 or Erastin, while the expression of H19 was dramatically decreased after treatment with the ferroptosis inhibitors Ferrostatin-1 or Liproxstatin-1 (Figure 1(a)). Erastin and Ferrostatin-1 were selected for subsequent experiments. The level of GSH in the OGD/H model group was significantly decreased, while iron content, LPO, and MDA levels were significantly increased (Figure 1(b–e)). In addition, erastin exposure was more potent in regulating the release of GSH, iron content, LPO, and MDA, whereas Ferrostatin-1 exerted the opposite effect (Figure 1(b–e)).

(a) Expression of H19 in BMVECs. (b) Levels of GSH in BMVECs. (c) Levels of iron in BMVECs. (d) Levels of LPO in BMVECs. (e) Levels of MDA in BMVECs.**P < 0.01, ***P < 0.001, compared with the control group. #*P* < 0.05, ##*P* < 0.01, compared with the OGD/H group.

### Knockdown of H19 promoted cell viability and inhibited ferroptosis of BMVECs

**Figure 2.**
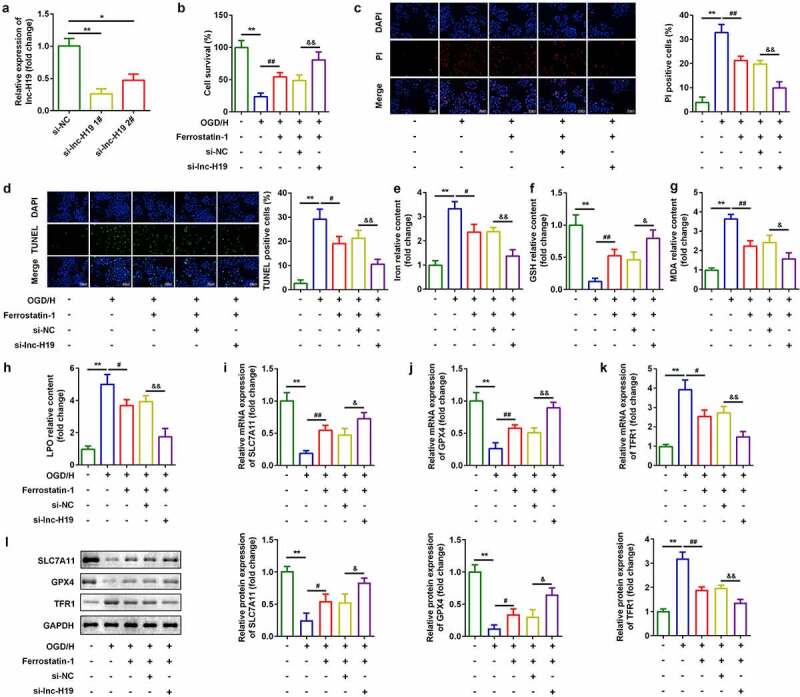
Knockdown of H19 promoted cell viability and inhibited ferroptosis

To investigate the roles of H19 in ICH, a siRNA specific for H19 was used to inhibit H19 expression in OGD/H-treated BMVECs. As shown in Figure 2(a), expression of H19 was decreased, which was more remarkable in the si-lncH19 1# group (Figure 2(a)). Inhibition of H19 improved cell viability (Figure 2(b)) and reduced cell death (Figure 2(c,d)). Simultaneously, after H19 was downregulated, levels of iron, MDA, and LPO decreased, while GSH content increased (Figure 2(e–h)). Furthermore, mRNA and protein expression of ferroptosis-related genes was detected. As shown in Figure 2(i–k), knockdown of H19 significantly increased mRNA expression of *SLC7A11* and *GPX4* and down-regulated *TFR1* levels (Figure 2(i–l)).

(a) H19 expression levels were detected using RT-qPCR after H19 knockdown. (b) Cell viability was measured using CCK8 assays after transfection with si-H19. (c) PI staining was used to detect cell death. (d) TUNEL staining was used to detect cell death. (e–h) Levels of iron, GSH, MDA, and LPO. (i–k) Expression of *SLC7A11, GPX4*, and *TFR1* mRNAs were measured using RT-qPCT. (l) Expression of SLC7A11, GPX4, and TFR1 proteins detected by western blotting.*P < 0.05, **P < 0.01, compared with si-NC group or control group. #*P* < 0.05, ##*P* < 0.01, compared with the OGD/H group. &*P* < 0.05, &&*P* < 0.01, compared with the OGD/H + Ferrostatin + si-NC group.

### H19 targeted miR-106b-5p in OGD/H BMVECs

**Figure 3.**
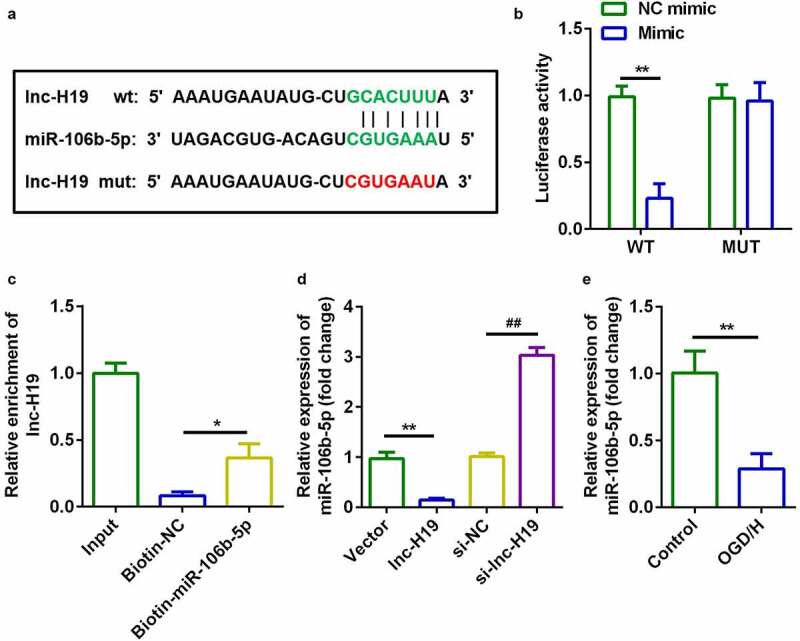
H19 sponged miR-106b-5p in BMVECs

Starbase v.3.0 (http://starbase.sysu.edu.cn/) was used to predict targets of H19. Figure 3(a) illustrates the binding sites between miR-106b-5p and H19. The luciferase activity of the luciferase-labeled miR-106b-5p mimic and wild-type H19 co-transfection groups was dramatically decreased compared to that of the NC mimic (Figure 3(b)). RNA pull-down assays further confirmed this interaction involving miR-106b-5p and H19 (Figure 3(c)). Furthermore, H19 downregulated the expression of miR-106b-5p in BMVECs, whereas knockdown of H19 resulted in the opposite effect (Figure 3(d)). Furthermore, miR-106b-5p expression was markedly decreased in OGD/H-treated BMVECs (Figure 3(e)). These data suggested that H19 targets miR-106b-5p in BMVECs.

(a) Bioinformatic analysis predicted the binding sites between miR-106b-5p and H19. (b) Dual-luciferase reporter assays confirmed that miR-106b-5p was a target of H19 in BMVECs. (c) Interactions between miR-106b-5p and H19 were determined using RNA pull-down assays. *P < 0.05 (d) The expression of miR-106b-5p was determined by RT-qPCR. (E) miR-106b-5p expression levels in BMVECs treated with or without OGD/H. *P < 0.05, **P < 0.01, ##*P* < 0.01.

### Down-regulation of miR-106b-5p reversed the effects of H19 on cell viability and ferroptosis

**Figure 4.**
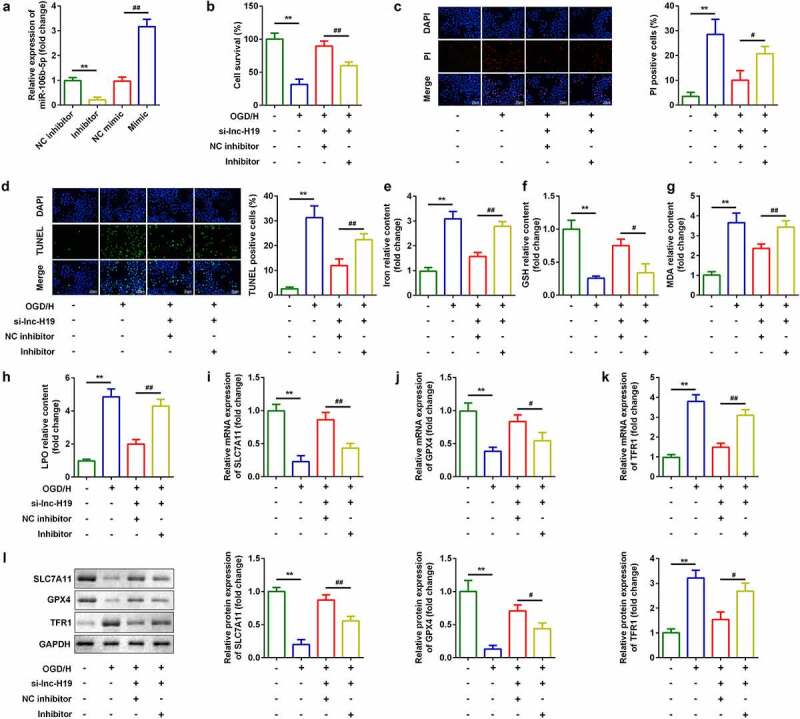
Down-regulation of miR-106b-5p reversed the effects of H19 knockdown on cell viability and ferroptosis of BMVECs

As shown in Figure 4(a), the expression of miR-106b-5p was sharply decreased in the miR-106b-5p inhibitor group (Figure 4(a)). Moreover, miR-106b-5p inhibitor suppressed cell viability (Figure 4(b)) and enhanced cell death (Figure 4(c,d)). Furthermore, downregulation of miR-106-5p reversed the regulatory roles in terms of the release of iron, MDA, LPO, and GSH (Figure 4(c–h)). Moreover, miR-106b-5p alleviated the effects of H19 knockdown on the expression of *SLC7A11, GPX4*, and *TRF1* at both the mRNA and protein levels (Figure 4(i–l)).

(a) miR-106b-5p expression was detected using RT-qPCR after H19`knockdown. (b) Cell viability was detected using CCK8 assays after transfection with si-H19. (c) PI staining was used to detect cell death. (d) TUNEL staining was used to detect cell death. (E-H) Levels of iron, GSH, MDA, and LPO. (i–k) Levels of *SLC7A11, GPX4*, and *TFR1* mRNAs were measured using RT-qPCT. (l) Expression of SLC7A11, GPX4, and TFR1 proteins detected by western blotting. **P < 0.01, compared with NC inhibitor group or control group. #*P* < 0.05, ##*P* < 0.01, compared with OGD/H + si-lnc-H19 + NC inhibitor group.

### ACSL4 was a target of miR-106b-5p

**Figure 5.**
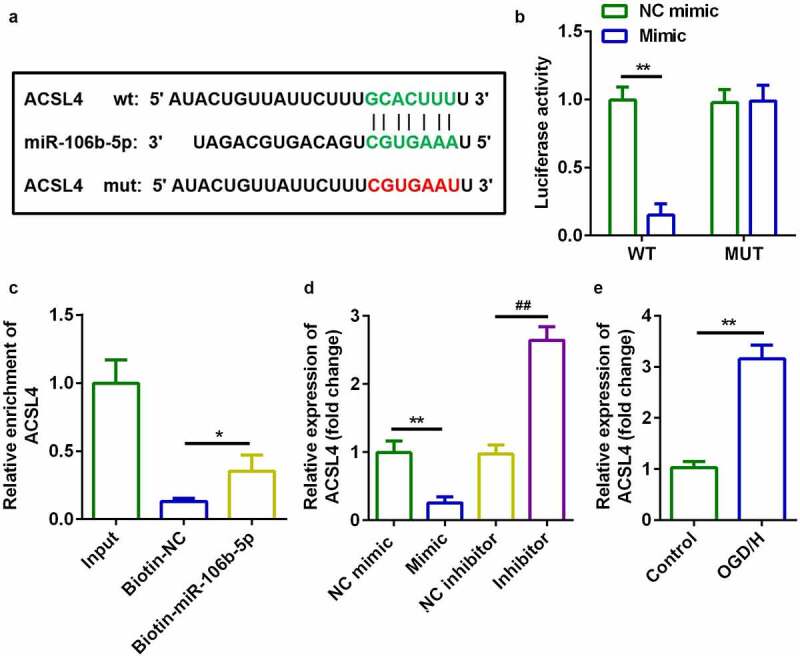
ACSL4 as a target gene of miR-106b-5p

TargetScan v.7.2 (http://www.targetscan.org/) indicated that acyl-CoA synthetase long-chain family member 4 (*ACSL4*) is a target gene of miR-106b-5p (Figure 5(a)). Luciferase activity of BMVECs was markedly decreased in cells transfected with miR-106b-5p mimic and wild-type ACSL4 3ʹ-UTR (Figure 5(b)). RNA pull-down assays further verified the interaction between ACSL4 and miR-106b-5p (Figure 5(c)). Additionally, mRNA expression of ACSL4 was significantly decreased by miR-106b-5p mimic and decreased by miR-106b-5p inhibitor (Figure 5(d)). Expression of ACSL4 mRNA was significantly increased in OGD/H-treated BMVECs (Figure 5(e)).

(a) Bioinformatic analysis predicted the binding sites between miR-106b-5p and *ACSL4*. (b) Dual-luciferase reporter gene assays confirmed that ACSL4 was a target of miR-106b-5p in BMVECs. (c) Interactions involving miR-106b-5p and ACSL4 were determined using RNA pull-down assays. (d) The expression of ACSL4 was determined by RT-qPCR. (e) ACSL4 expression levels in BMVECs with or without treatment by OGD/H.*P < 0.05, **P < 0.01, ##*P* < 0.01.

### Up-regulation of ACSL4 inhibited the effects of miR-106b-5p

**Figure 6.**
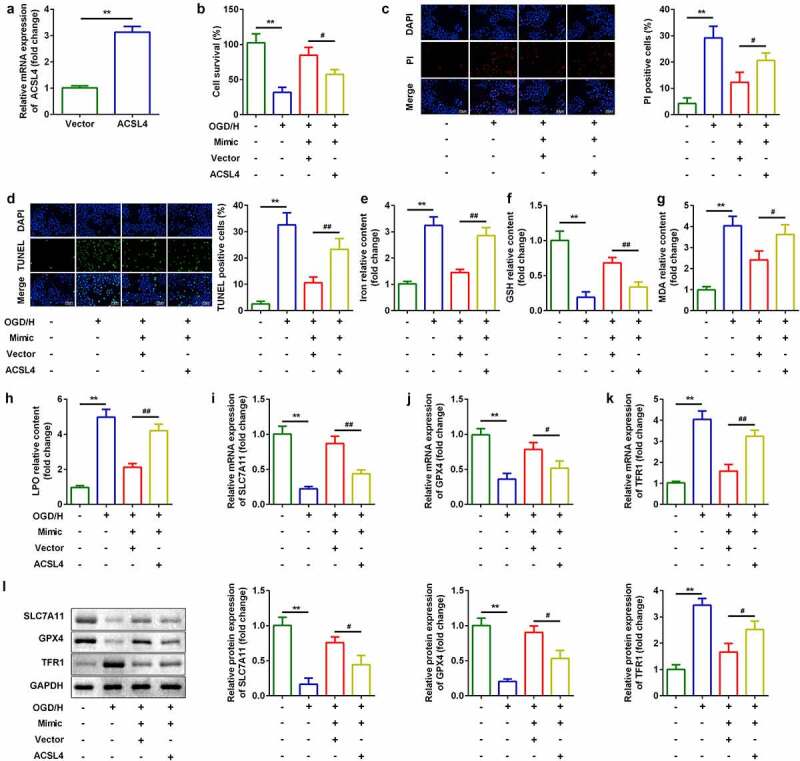
Up-regulation of ACSL4 inhibited the effects of miR-106b-5p

Considering the effects of ACSL4 on miR-106b-5p, we overexpressed ACSL4 in miR-106b-5p up-regulated cells (Figure 6(a)). Compared with miR-106b-5p-overexpressing cells, co-transfection with miR-106b-5p and ACSL4 vector inhibited cell viability (Figure 6(b)) and accelerated ferroptosis (Figure 6(c–l)).

(a) ACSL4 expression was detected using RT-qPCR after miR-106b-5p overexpression. (b) Cell viability was detected using CCK8 assays after transfection with miR-106b-5p mimic. (c) PI staining was used to detect cell death. (d) TUNEL staining was used to detect cell death. (e–h) Levels of iron, GSH, MDA, and LPO. (i–k) Expression of *SLC7A11, GPX4*, and *TFR1* mRNAs were measured using RT-qPCT. (l) Expression of SLC7A11, GPX4, and TFR1 proteins detected by western blotting.**P< 0.01, compared with vector group or control group. #*P* < 0.05, ##*P* < 0.01, compared with the OGD/H + mimic + vector group.

## Discussion

ICH is a cerebrovascular disease with high disability and mortality rates [1,2]. At present, it is only treated with surgery and primary care and lacks specific therapeutic targets [2]. Thus, there is an urgent requirement to identify exact therapeutic targets for ICH therapy. Recent evidence suggests that lncRNAs may be associated with ICH occurrence. For instance, the lncRNA *NKILA* is differentially expressed in ICH and protects against brain injuries [30]. The lncRNA *FENDRR* is upregulated in ICH and leads to the attenuation of brain injuries [31]. Moreover, lncRNA *Blnc1* is a potential biological marker for ICH [32]. Our data demonstrated that lncRNA H19 suppressed cellular proliferation and promoted BMVEC ferroptosis in ICH by regulating the miR-106b-5p/ACSL4 axis.

Many studies indicate that lncRNA H19 is involved in the pathogenesis of several human tumors [33–35]. H19 also plays a specific regulatory role in diabetic retinopathy, atherosclerosis, and ankylosing spondylitis [36–38]. H19 levels are significantly higher in ICH rat models than in normal rats [21]. Thus, H19 may be a biomarker of ICH. Bai et al. demonstrated that H19 may affect ferroptosis in spontaneous abortion [39] in this study, H19 was upregulated in ICH injuries *in vivo* and *in vitro*, which was in accord with previous studies [40]. Suppression of H19 in OGD/H ICH model cells promoted cell viability and inhibited ferroptosis.

lncRNAs function as sponges for miRNAs and regulate cellular processes by reducing their expression. To further investigate the explicit regulatory mechanisms involved, we performed predictive analysis and found that miR-106b-5p was a target miRNA of H19. miR-106b-5p is abnormally expressed in various cancers [41]. Recent studies imply that miR-106b-5p may be used as a diagnostic biomarker for acute stroke and cerebral ischemic events [42,43]. Nevertheless, miR-106b-5p has not been investigated in ICH. miR-106b-5p is a target of H19, and miR-106b-5p expression was decreased in OGD/H ICH model cells In addition, downregulation of miR-106b-5p reversed the effects of H19 on cell viability and ferroptosis. In this study, H19 knockdown may protect against ICH by binding to miR-106b-5p.

ACSL4 is a crucial regulator of ferroptosis [44]. Knockdown of ACSL4 has been reported to provide a new therapeutic approach for ischemic stroke by inhibiting ferromyopathy-induced brain damage and neuroinflammation [45]. In this study, ACSL4 was the target gene of miR-106b-5p and was upregulated in OGD/H ICH model cells. Overexpression of ACSL4 reversed the effect of miR-106b-5p, inhibited ICH cell viability, and promoted ferroptosis. These results suggested that ACSL4 may function as a ferroptosis promoter and degrade the cellular functions of BMVECs, which is consistent with a study by Xie et al. [32]. Moreover, H19 acted as a (competing endogenous RNA) ceRNA to regulate the proliferation and ferroptosis of BMVECs via the miR-106b-5p/ACSL4 axis.

## Conclusions

Taken together, H19 knockdown protected against ICH by regulating miR-106b-5p/ACSL4. Therefore, the H19/miR-106b-5p/ACSL4 axis may represent an alternative therapeutic target for the treatment of ICH.

## Highlights

- lncRNA H19 was up-regulated in OGD/H ICH model cells.
- Knockdown of lncRNA H19 promoted cell viability and inhibited cell ferroptosis.
- miR-106b-5p was sponged by lncRNA H19, and miR-106b-5p directly targeted ACSL4 to participate in the regulation of ICH injury.
